# The Cognitive Effects of Antidepressants in Major Depressive Disorder: A Systematic Review and Meta-Analysis of Randomized Clinical Trials

**DOI:** 10.1093/ijnp/pyv082

**Published:** 2015-07-25

**Authors:** Joshua D Rosenblat, Ron Kakar, Roger S McIntyre

**Affiliations:** Mood Disorder Psychopharmacology Unit, University Health Network, Department of Psychiatry, University of Toronto, Canada (Drs Rosenblat, Kakar, and McIntyre); Departments of Psychiatry and Pharmacology, University of Toronto, Toronto, Canada (Drs Rosenblat and McIntyre); Department of Psychiatry, Western University, London, and Windsor, Ontario, Canada (Dr Kakar).

**Keywords:** antidepressants, cognitive function, executive function, major depressive disorder, psychomotor speed, working memory

## Abstract

**Background:**

Cognitive dysfunction is often present in major depressive disorder (MDD). Several clinical trials have noted a pro-cognitive effect of antidepressants in MDD. The objective of the current systematic review and meta-analysis was to assess the pooled efficacy of antidepressants on various domains of cognition in MDD.

**Methods:**

Trials published prior to April 15, 2015, were identified through searching the Cochrane Central Register of Controlled Trials, PubMed, Embase, PsychINFO, Clinicaltrials.gov, and relevant review articles. Data from randomized clinical trials assessing the cognitive effects of antidepressants were pooled to determine standard mean differences (SMD) using a random-effects model.

**Results:**

Nine placebo-controlled randomized trials (2 550 participants) evaluating the cognitive effects of vortioxetine (n = 728), duloxetine (n = 714), paroxetine (n = 23), citalopram (n = 84), phenelzine (n = 28), nortryptiline (n = 32), and sertraline (n = 49) were identified. Antidepressants had a positive effect on psychomotor speed (SMD 0.16; 95% confidence interval [CI] 0.05–0.27; I^2^ = 46%) and delayed recall (SMD 0.24; 95% CI 0.15–0.34; I^2^ = 0%). The effect on cognitive control and executive function did not reach statistical significance. Of note, after removal of vortioxetine from the analysis, statistical significance was lost for psychomotor speed. Eight head-to-head randomized trials comparing the effects of selective serotonin reuptake inhibitors (SSRIs; n = 371), selective serotonin and norepinephrine reuptake inhibitors (SNRIs; n = 25), tricyclic antidepressants (TCAs; n = 138), and norepinephrine and dopamine reuptake inhibitors (NDRIs; n = 46) were identified. No statistically significant difference in cognitive effects was found when pooling results from head-to-head trials of SSRIs, SNRIs, TCAs, and NDRIs. Significant limitations were the heterogeneity of results, limited number of studies, and small sample sizes.

**Conclusions:**

Available evidence suggests that antidepressants have a significant positive effect on psychomotor speed and delayed recall.

## Introduction

Major depressive disorder (MDD) is a highly prevalent and disabling illness affecting greater than 350 million people worldwide (Kessler et al., 2006). The World Health Organization (2012) has recognized MDD as the leading cause of disability, causing significant and often chronic functional impairment. Cognitive dysfunction associated with MDD is a key feature sub-serving the functional impairment associated with MDD (Baune et al., 2010). Several cognitive domains, including executive function, attention, memory, processing speed, and psychomotor skills, are affected during both symptomatic as well as “remitted” phases in MDD (Marazziti et al., 2010; Lee et al., 2012; Bora et al., 2013; Bortolato et al., 2014). Given the significant and persistent functional impairment mediated by cognitive dysfunction, increased attention is being given to this domain in the treatment of MDD (Bortolato et al., 2014).

Several investigators have studied the cognitive effects of various antidepressants (Keefe et al., 2014); however, the majority of these studies were limited by small sample sizes, absence of placebo controls, a lack of pre-specification of cognition as a primary outcome, and insufficient statistical analytic approaches to parse direct versus indirect effects (e.g. path analysis; McIntyre et al., 2013). Many studies have reported a positive effect of various antidepressants on cognition, yielding a statistically significant difference between groups receiving treatment versus placebo; however, quantification of the overall and relative magnitude of effect (e.g. the pooled standard mean difference [SMD]) of all currently available antidepressants on cognition has yet to be conducted. Notably, Keefe et al. (2014) conducted a systematic review on the cognitive effects of pharmacotherapy in MDD, in which they calculated the effect size for all studies reviewed; however, they did not meta-analytically quantify pooled effect sizes of the cognitive effects of antidepressants. In addition, since the publication of their review, several new clinical trials have been published that have primarily sought to determine the effects of antidepressants on cognitive function (Katona et al., 2012; McIntyre et al., 2014; Robinson et al., 2014; Soczynska et al., 2014; Gorlyn et al., 2015; Mahableshwarkar et al., 2015).

Therefore, the primary objective of the current systematic review and meta-analysis is to assess the overall effect of antidepressants on cognition in MDD as determined in placebo-controlled trials. As a secondary objective, the relative efficacy of mechanistically diverse antidepressants on cognitive function will be compared based on effect sizes calculated from comparative head-to-head trials. The pertinence of this review is two-fold: (1) given that cognition is becoming an increasingly important target in the treatment of MDD, knowledge regarding the effect size of currently available therapies is essential; and (2) with the increased pursuit of novel therapeutic strategies targeting cognition in MDD, a benchmark of effect size should be established.

## Methods

### Search Methods for Identification of Trials

The PubMed, PsycInfo, Cochrane, and Embase databases were searched from inception to April 15, 2015. The PubMed search was limited to human studies, including clinical trials, observational studies, meta-analyses, and review articles written in the English language using the following search string: (major depressive disorder OR unipolar depression) AND (cognitive function OR cognitive impairment OR cognitive dysfunction OR executive function OR executive dysfunction OR memory OR attention). Various combinations of additional search terms were used to search for additional articles in all four databases (search terms listed in Supplementary Material). Reference lists from identified articles were manually searched for additional relevant studies. All identified articles were screened by two independent reviewers (Drs Rosenblat and Kakar) for inclusion in qualitative and quantitative analyses. Where there was disagreement on inclusion, consensus was reached through discussion.

### Inclusion Criteria

1. Human studies with participants over the age of 18 (no upper limit) with a diagnosis of MDD as defined by the Diagnostic and Statistical Manual or International Classification of Disease criteria (no restrictions on edition used);
2. Randomized clinical trial of antidepressants with the primary mechanism of action being monoamine modulation in one or more of the following categories: selective serotonin reuptake inhibitors (SSRIs), selective serotonin and norepinephrine reuptake inhibitors (SNRIs), norepinephrine and dopamine reuptake inhibitors (NDRIs), serotonin antagonist and reuptake inhibitors, noradrenergic and specific serotonergic antidepressants, tricyclic antidepressants (TCAs), and multimodal antidepressants (e.g. vortioxetine);
3. Cognition was assessed using standardized and validated measures;
4. Data was provided to allow for calculation of effect size (where insufficient data was provided in the article, the authors were contacted to obtain the required data); and
5. Manuscript is written in English

### Exclusion Criteria

Excluded study descriptions and reasons for exclusion are summarized in Supplemental Table 1.

1. Unpublished data or conference abstracts;
2. Open-label trials and observational studies;
3. Studies using healthy controls, instead of placebo-controlled MDD patients, to determine effect (some of these studies are discussed in the qualitative analysis, but were not included in the quantitative analysis);
4. Clear methodological flaws, such as lack of randomization or large variance in treatment and placebo groups baseline characteristic and/or psychometric measures (included in qualitative review but not quantitative analysis);
5. Multiple reports from the same data set (e.g. only original study was included to prevent overweighting of one data set);
6. Studies explicitly including participants with other psychiatric or neurologic diagnoses such as bipolar disorder, schizophrenia, schizoaffective disorder, attention deficit hyperactivity disorder, or dementia;
7. Studies explicitly including participants using concomitant choline esterase inhibitors or stimulants; and
8. While studies using TCAs were included, trials assessing the effects of tianeptine were excluded, as the primary mechanism of action of tianeptine is now believed to be via glutamate modulation (Nickel et al., 2003).

### Data Extraction and Statistical Analysis

Using standardized data extraction forms, data was extracted from included studies by two independent reviewers (Drs Rosenblat and Kakar) to systematically evaluate study characteristics, risks of bias, and cognitive testing results required for the calculation of effect size. Final cognitive scores of treatment versus placebo were used for the analysis, as recommended by the *Cochrane Handbook for Systematic Review of Interventions*, except where large pre-treatment differences in cognitive scores were identified; for these studies the change from baseline was compared instead to prevent skewing of results. Where mean and/or standard deviation values were not reported, these were calculated based on reported confidence intervals (CI) or *p*-values. Where inadequate information was reported to calculate mean and standard deviations, values essential for determining Cohen’s d effect size/SMD, the study authors were contacted directly for this additional data. For two studies (Georgotas et al., 1989; Raskin et al., 2007), only means were reported and the original authors could not provide standard deviation values. For these studies, the average standard deviation was extrapolated from other studies using the same cognitive test and was utilized for SMD calculations.

Pooling of effect sizes and tests of heterogeneity were conducted using Review Manager 5.3 (Copenhagen: The Nordic Cochrane Centre, The Cochrane Collaboration) software using a random-effects model. Effect sizes, using Cohen’s d effect size, where 0.2 = small, 0.5 = medium, and 0.8 = large, were calculated using SMD in post-treatment neuropsychological performance between antidepressant treatment and placebo, for placebo-controlled trials, and antidepressant compared to another antidepressant, for comparative head-to-head trials of different antidepressants. Samples were not sub-grouped into responders and non-responders, as an insufficient number of studies reported responder sub-grouped analysis; rather, the mean effect for all subjects was included for effect size calculations.

Neuropsychological testing from included studies was pooled based on the cognitive domain being tested (see Strauss and Spreen, 2006, for a review of cognitive tests and domains). Results for placebo-controlled trials were only pooled for domains wherein two or more studies evaluating the same domain were identified. In placebo-controlled trials with multiple antidepressant groups, separate effect sizes were calculated with respect to the one common placebo control group. Pooled effect sizes were calculated separately for each antidepressant, then pooled to calculate the overall effect size of all antidepressants included. Individual agents or studies were subsequently removed from the pooled sample to determine if removal of any one specific agent or study could significantly alter the overall effect size. In addition, for trials assessing psychomotor speed, an additional subgroup analysis was conducted, separating studies including subjects with a mean age of less than versus greater than 65 years.

For studies directly comparing antidepressants’ relative effects on cognition without a placebo group, effect size was determined in a similar manner, except by replacing the placebo group with the comparator antidepressant, effectively determining an effect size in relation to the first antidepressant.

Critical values for pooled effect sizes were set at 0.05. Homogeneity in effect sizes was tested using the Q statistic (Chi^2^) for each cognitive domain and each antidepressant. Heterogeneity was quantified using the I^2^ statistic, where 25% = small, 50% = moderate, and 75% = high heterogeneity (Higgins et al., 2003).

### Assessment of Bias

The risk of bias was assessed for all clinical trials included in the quantitative analysis. As per recommendations in the *Cochrane Handbook for Systematic Review of Interventions*, bias was assessed based on the following five domains: sequence generation (e.g. based on description of randomization), allocation concealment, blinding of outcome assessors, intention-to-treat, and for-profit bias. Risk of bias was designated to be high if described protocols were concerning for bias in a given domain or if description of the domain was omitted from the primary text and primary authors could not provide clarification when contacted. For example, if sequence generation methods were not explicitly described and the study author could not provide clarification when contacted, this domain would be labeled as high risk. Where an adequate protocol was described for a given domain, it would be labeled low risk.

To assess publication bias, a funnel plot was created using Review Manager 5.3 Software for forest plots with greater than five studies included. An Egger Test was not conducted, as greater than ten studies are required in accordance with the *Cochrane Review Handbook*; the current analysis had a maximum of nine studies included in any given forest plot.

## Results

### Search Results and Study Characteristics

**Figure 1.**
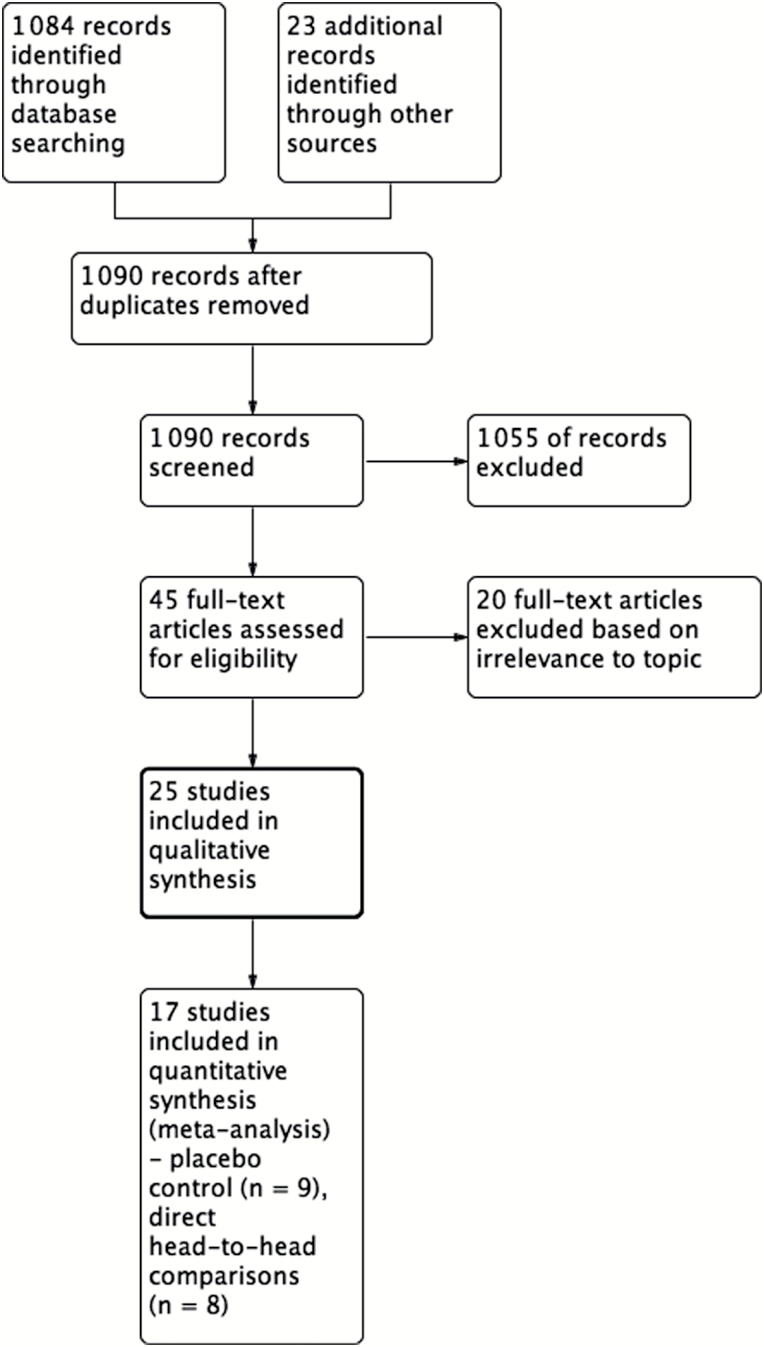
Preferred Reporting Items for Systematic Reviews and Meta-Analyses flow diagram of study selection.

Electronic database searches yielded a total of 1 084 articles (Figure 1). A manual review of reference lists and suggested studies from experts in the field revealed an additional 23 potentially relevant articles. Titles and abstracts were screened, yielding 45 articles for which the full text was reviewed for inclusion. Of these studies, 25 were found appropriate to be included in the qualitative review, of which 17 were included for the quantitative analysis. Demographic information, the antidepressant studied, and cognitive testing for each study included in the quantitative analysis are summarized in Table 1.

**Table 1.** Demographic Information and Study Design of Included Placebo-Controlled Trials

Study names	Study Length (weeks)	Diagnostic Criteria	Age (range, mean ± SD) Sex (% female)	Treatment Group (n)	Cognitive Testing	Effect (+/-/neutral), statistically NS
McIntyre et al., 2014	8	-DSM IV -TR -MDD -MADRS ≥ 26 -Current MDE ≥ 3 months	18–65 45.4±12.2 68.7% 45.6±12.1 65.8%	Vortioxetine 10mg (192) vs Placebo (194)	Composite z-score [DSST/ RAVLT(acq)/RAVLT (delay)] DSST(correct Symbols) RAVLT(acquisition) RAVLT(delayed recall) TMT-A TMT-B SRT CRT Stroop(congruent) Stroop(incongruent) PDQ(total score)	+ve *p* < 0.001 +ve *p* < 0.001 +ve *p* = 0.029 +ve *p* = 0.003 +ve *p* = 0.006 +ve *p* = 0.006 +ve *p* < 0.001 +ve *p* < 0.001 +ve *p* = 0.002 +ve *p* = 0.001 +ve *p* < 0.001
			46.1±11.8 64.3% 45.6±12.1 65.8%	Vortioxetine 20mg (204) vs Placebo (194)	Composite z-score [DSST/ RAVLT(acq)/RAVLT (delay)] DSST(correct symbols) RAVLT(acquisition) RAVLT(delayed recall) TMT-A TMT-B SRT CRT Stroop(congruent) Stroop(incongruent) PDQ(total score)	+ve *p* < 0.001 +ve *p* < 0.001 +ve *p* = 0.199 +ve *p* = 0.007 +ve *p* = 0.005 +ve *p* < 0.001 +ve *p* = 0.016 +ve *p* = 0.355 +ve *p* < 0.001 +ve *p* = 0.001 +ve *p* < 0.001
Katona et al., 2012	8	-DSM IV -TR -MDD -MADRS ≥ 26 -Current MDE ≥ 4wks (at least 2nd episode)	65+ 70.5±4.8 68.6% 70.3±4.4 62.1%	Vortioxetine 5mg (156) vs Placebo (145)	DSST(correct symbols) RAVLT(acquisition) RAVLT(delayed recall)	+ve *p* < 0.05 +ve *p* < 0.05 +ve *p* < 0.05
			70.9±5.5 66.2% 70.3±4.4 62.1%	Duloxetine 60mg (151) vs Placebo (145)	DSST(correct symbols) RAVLT(acquisition) RAVLT(delayed recall)	Neutral NS +ve *p* < 0.01 +ve *p* < 0.01
Mahableshwarkar et al., 2015	8	-DSM IV -TR -MDD -MADRS ≥ 26 -Recurrent MDD with current MDE	18–65 44.2±12.2168.2% 45.0±12.0761.3%	Vortioxetine10-20mg (175) vs Placebo (167)	DSST(correct symbols) PDQ TMT-A TMT-B Stroop(congruent) Stroop(incongruent)	+ve *p* = 0.019 +ve *p* = 0.001 +ve *p* = 0.446 +ve *p* < 0.001 +ve *p* = 0.482 +ve *p* = 0.980
			45.7±11.4665.7% 45.0±12.0761.3%	Duloxetine 60mg (187) vs Placebo (167)	DSST(correct symbols) PDQ TMT-A TMT-B Stroop(congruent) Stroop(incongruent)	+ve *p* = 0.099 +ve *p* < 0.001 +ve *p* = 0.303 +ve *p* = 0.053 +ve *p* = 0.904 +ve *p* = 0.422
Raskin et al., 2007	8	-DSM IV -MDD -HAMD(17) ≥18 -Recurrent MDD with current MDE	65+ 72.6±5.7 60.4% 73.3±5.7 57.7%	Duloxetine 60mg (196) vs Placebo (99)	Verbal learning and recall test (learning trials) (delayed recall) DSST(correct symbols) Two-digit cancellation test Letter-number sequencing test	+ve *p* = 0.03 +ve *p* = 0.02 Neutral NS Neutral NS Neutral NS
Robinson et al., 2014	24	-DSM IV -TR -MDD -MADRS ≥ 20 -Current MDE	65+ 73.01±6.2666.2 73.1±5.64 58.9%	Duloxetine 60 or 120mg (180) Placebo (87)	Verbal learning and recall test (learning trials) (delayed recall) DSST(correct symbols) Two-digit cancellation test TMT-B	Neutral NS Neutral NS Neutral NS Neutral NS Neutral NS
Culang et al., 2009	8	-DSM IV-MDD -HRSD(24) ≥ 20 -Curent MDE	75+ 79.82±3.9754% 79.33±4.6962%	Citalopram 20mg (40mg if HRSD > 10 after 4 wks) (84) Placebo (90)	DSST Stroop CRT JOLO Buschke SRT	Neutral NS Neutral NS Neutral NS Neutral NS Neutral NS
Ferguson et al., 2003	8	-DSM IV -MDD -HAM-D(17) ≥ 20	18–65 Not available	Paroxetine 20–40mg (23) vs Placebo (26)	Continuity of Attention Combined speed	Neutral NS Neutral NS
Georgotas et al., 1989	7	-MDD Diagnosis^a^ -HRSD(21) ≥ 16	55+ 64.7±6.8 55.1% (age for all groups totaled)	Nortriptyline (32) vs Phenelzine (28) vs Placebo (18) (Dosing not specified)	Paragraph recall Paragraph recall delay Paired associated recall WAIS Vocobularly DSST Mental Status Questionaire Name-Face Test Peterson and Peterson Finger tapping Buschke Retrieval Task	Neutral NS Neutral NS Neutral NS Neutral NS Neutral NS Neutral NS Neutral NS Neutral NS Neutral NS Neutral NS
Hoffman et al., 2008	16	-DSM IV- MDD -BDI-II ≥ 12 -HAM-D(29)	40+ 51.8 ± 7.7 75.5% 51.2 ± 7.8 77.5%	Sertraline 50–200mg (49) Placebo (49)	Logical memory Verbal pairs, easy Verbal pairs, hard Digits forward Digits Backwards Animal naming COWAT Stroop Ruff total DSST TMT-B	Neutral NS Neutral NS Neutral NS Neutral NS Neutral NS Neutral NS Neutral NS Neutral NS Neutral NS Neutral NS Neutral NS

^a^MDD diagnosed by research diagnostic criteria

BDI-II, Beck Depression Inventory-II; Buschke SRT, Buschke Selective Reminding Test; COWAT, Wechsler Adult Intelligence Scale, Controlled Oral Word Association Test; CRT, Cognitive Reflection Test; DSM-IV, Diagnostic and Statistical Manual of Mental Disorders; DSST, Digit Symbol Sign Test; HAM-D, Hamilton Rating Scale For Depression; HRSD, Hamilton Rating Scale For Depression; JOLO, Judgment of Line Orientation; MADRS, Montgomery-Asberg Depression Rating Scale; MDD, Major Depressive Disorder; MDE, Major Depressive Episode; NS, non-significant; PDQ, Perceived Deficit Questionnaire; RAVLT, Rey Auditory Verbal Learning Test; SD, standard deviation SRT, Simple Reaction Time; TMT-A or -B, Trails Making Test; TR, text revision; WAIS, Wechsler Adult Intelligence Scale.

Of the articles included in the quantitative review, nine were placebo-controlled randomized trials, with four trials evaluating two antidepressants compared to the same placebo group and the remaining evaluating a single antidepressant compared to placebo. Of the placebo-controlled trials, studies evaluated the cognitive effects of vortioxetine (n = 3), duloxetine (n = 4), paroxetine (n = 1), citalopram (n = 1), phenelzine (n = 1), nortryptiline (n = 1), and sertraline (n = 1). Four studies compared TCAs (notriptyline, desipramine, dotheipin) to SSRI/SNRIs (sertraline, fluoxetine, venlafaxine). Two studies compared NDRIs (bupropion) to SSRIs (paroxetine and escitalopram). Two studies directly compared sertraline and fluoxetine.

In addition to the studies included in the quantitative analysis, eight studies were identified for qualitative review. These clinical trials were excluded from the quantitative analysis due to their study design (observational studies, open label studies, lack of placebo controls, or appropriate comparative group) and/or agents used (e.g. non-monoaminergic agents); however, these studies were still deemed to be noteworthy within the scope of this review and are summarized separately in Supplementary Table 1.

### Domains of Cognition Evaluated

Table 1 summarizes cognitive tests used in included studies. Pooled SMD were only calculated where two or more studies were included the same domain. As such, the domains that allowed for a meaningful determination of pooled SMD were psychomotor speed (evaluated by digit symbol sign test [DSST] and combined speed testing), cognitive control (evaluated by Stroop test), executive function (evaluated by Trails Making Test-B [TMT-B]), and delayed recall (evaluated by Rey Auditory Verbal Learning Test [RAVLT]) for placebo-controlled trials. For head-to-head comparative trials, results were pooled for domains of memory (shopping list task, Burke SRT, Rivermead Behavioral Memory Test, and Kim’s game) and working memory.

### Pooled Effect Sizes for Placebo-Controlled Trials

#### Effect on Psychomotor Speed (DSST or Combined Speed)

**Figure 2.**
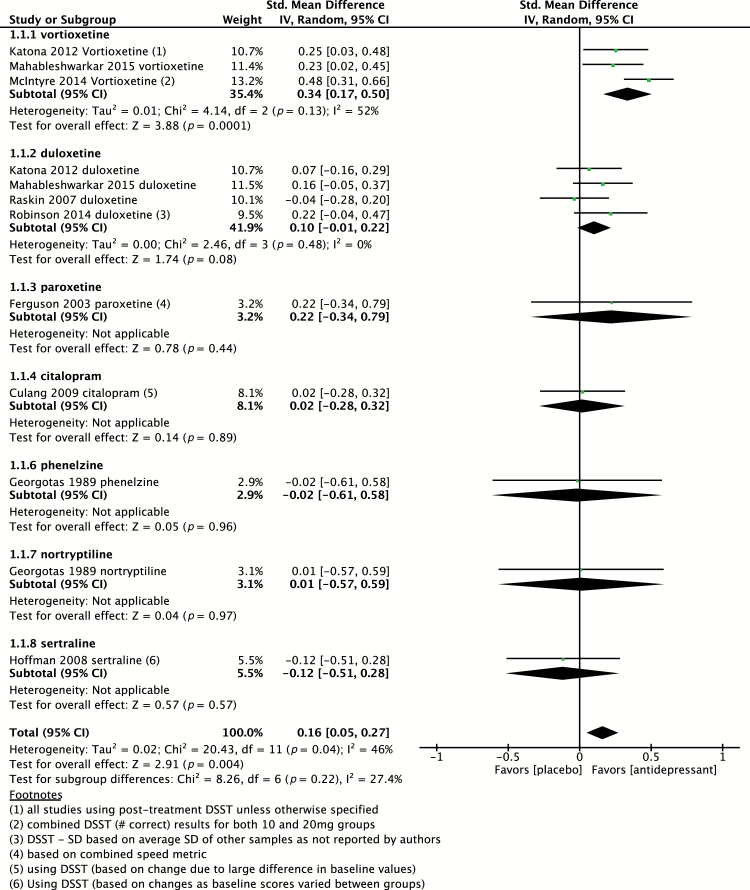
Pooled effect for placebo-controlled trials assessing psychomotor speed. CI, confidence interval; DSST, Digit Symbol Sign Test; SD, standard definition.

In total, nine placebo-controlled studies evaluated psychomotor speed using DSST or a combined speed measure (Georgotas et al., 1989; Ferguson et al., 2003; Raskin et al., 2007; Hoffman et al., 2008; Culang et al., 2009; Katona et al., 2012; McIntyre et al., 2014; Robinson et al., 2014; Mahableshwarkar et al., 2015). Of these studies, three studies evaluated two agents in parallel compared to placebo, providing a total of twelve independent effect sizes to pool, including evaluation of vortioxetine (n = 3), duloxetine (n = 4), paroxetine (n = 1), citalopram (n = 1), phenelzine (n = 1), nortryptiline (n = 1), and sertraline (n = 1). As shown in Figure 2, the pooled effect size of all antidepressants (n = 1 660) versus placebo (n = 875) was 0.16 (95% CI 0.05 to 0.27; *p* = 0.004), indicative of a small, yet statistically significant, positive effect. Heterogeneity was found to be moderate, with I^2^ = 46% (*p* = 0.04).

Of the antidepressants evaluated, vortioxetine (n = 728) had the largest pooled effect size, of 0.34 (95% CI 0.17 to 0.50; *p* = 0.0001), as compared to 0.10 (95% CI -0.01 to 0.22) for duloxetine (n = 498), 0.22 (95% CI -0.34 to 0.79) for paroxetine (n = 23), 0.02 (95% CI -0.28 to 0.32) for citalopram (n = 84), 0.02 (95% CI -0.28 to 0.32) for sertraline (n = 49), -0.02 (95% CI -0.61 to 0.58) for phenelzine (n = 28), and 0.01 (95% CI -0.57 to 0.59) for nortyptiline (n = 32).

Of interest, when removing vortioxetine from the pooled SMD, the effect size was no longer statistically significant compared to placebo (SMD 0.08; 95% CI -0.02 to 0.18; *p* = 0.13) and the heterogeneity was small (Chi^2^ = 4.10; *p* = 0.85; I^2^ = 0%). Also, with the removal of TCAs, the pooled effect size remained unchanged.

**Figure 3.**
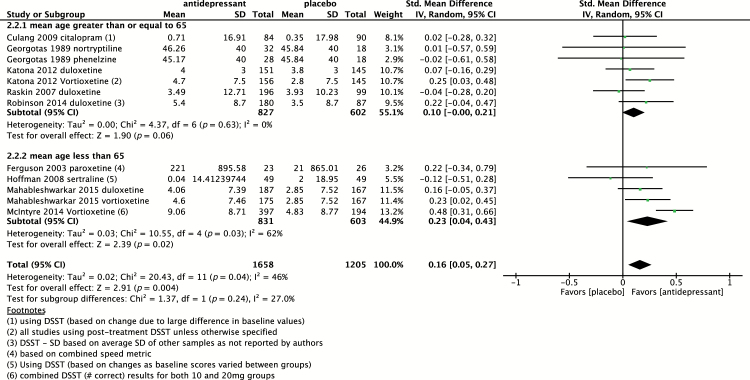
Pooled effect of placebo-controlled trials assessing psychomotor speed sub-grouped based on age greater or less than 65 years. CI, confidence interval; DSST, Digit Symbol Sign Test; SD, standard definition.

**Figure 4.**
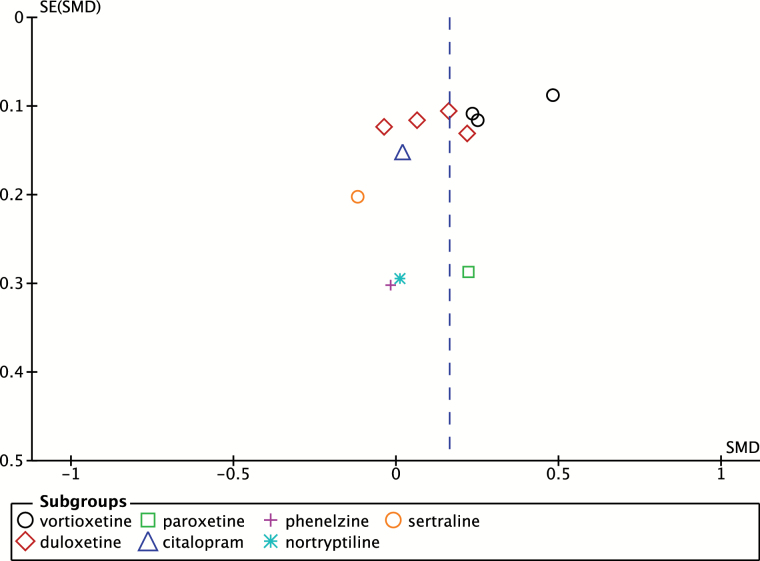
Funnel-plot of placebo-controlled trials assessing psychomotor speed. SE, standard error; SMD, standard mean differences.

A subgroup analysis comparing studies with subjects with a mean age greater than 65 versus less than 65 was also conducted, as shown in Figure 3. For studies with subjects older than 65, the SMD was 0.10 (95% CI 0.00 to 0.21; *p* = 0.06) as compared to 0.23 (95% CI 0.04 to 0.43; *p* = 0.02) in subjects younger than 65, suggestive of a greater positive effect in subjects under 65; however, the difference between subgroups was not statistically significant (*p* = 0.24). A funnel plot to assess for publication bias was also conducted, as shown in Figure 4.

#### Effect on Cognitive Control (Stroop Test)

**Figure 5.**
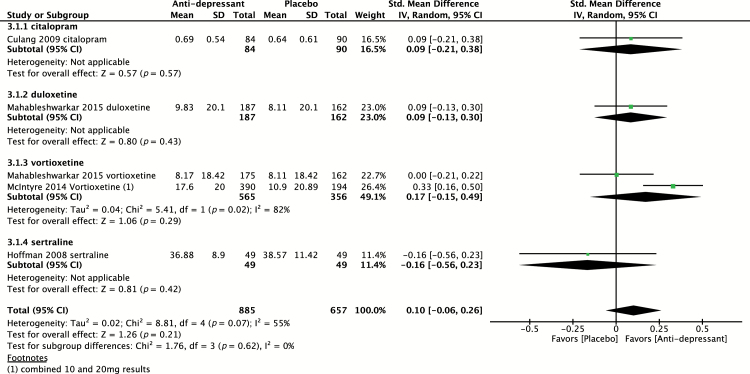
Pooled effect for placebo-controlled trials assessing cognitive control (Stroop test). CI, confidence interval; SD, standard definition.

Four placebo-controlled trials (Hoffman et al., 2008; Culang et al., 2009; McIntyre et al., 2014; Mahableshwarkar et al., 2015) evaluated the effect of antidepressants on cognitive control using the Stroop test. Of these studies, one study (Mahableshwarkar et al., 2015) evaluated two agents in parallel compared to placebo, providing a total of five independent effect sizes to pool, including evaluation of vortioxetine (n = 2), duloxetine (n = 1), citalopram (n = 1), and sertraline (n = 1). The pooled effect size of all antidepressants (n = 885) versus placebo (n = 494) was 0.10 (95% CI -0.06 to 0.26; *p* = 0.21) indicative of a non-statistically significant effect (Figure 5). Heterogeneity was found to be moderate, with I^2^ = 55% (*p* = 0.07).

#### Effect on Executive Function (TMT-B)

**Figure 6.**
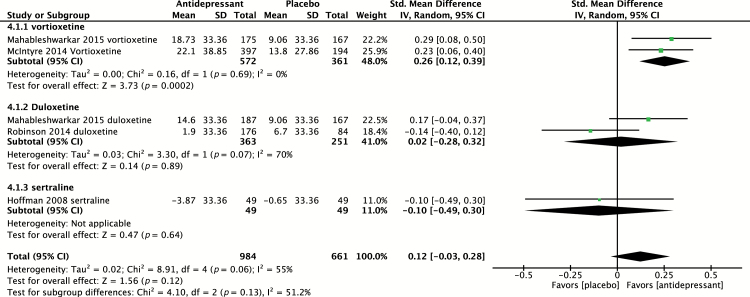
Pooled effect for placebo-controlled trials assessing executive function (Trails Making Test-B). CI, confidence interval; SD, standard definition.

Four placebo-controlled trials (Hoffman et al., 2008; McIntyre et al., 2014; Robinson et al., 2014; Mahableshwarkar et al., 2015) evaluated the effect of antidepressants on executive function using TMT-B. Of these studies, one study (Mahableshwarkar et al., 2015) evaluated two agents in parallel compared to placebo, providing a total of five independent effect sizes to pool, including evaluation of vortioxetine (n = 2), duloxetine (n = 2), and sertraline (n = 1). The SMD of all antidepressants (n = 984) versus placebo (n = 494) was 0.12 (95% CI -0.03 to 0.28; *p* = 0.12) is shown in Figure 6, indicative of a non–statistically significant effect. Heterogeneity was found to be moderate, with I^2^ = 55% (*p* = 0.06).

#### Effect on Delayed Recall

**Figure 7.**
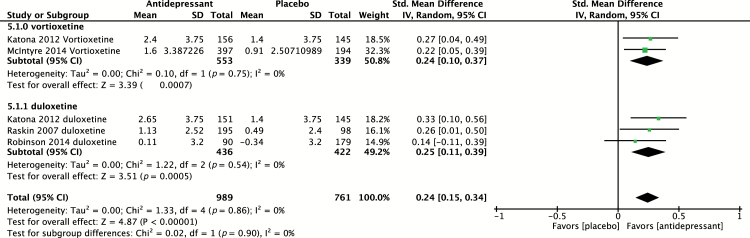
Pooled effect for placebo-controlled trials assessing delayed recall (Rey Auditory Verbal Learning Test). CI, confidence interval; SD, standard definition.

Four placebo-controlled trials (Raskin et al., 2007; Katona et al., 2012; McIntyre et al., 2014; Robinson et al., 2014) evaluated the effect of antidepressants on delayed recall using RAVLT. Of these studies, one study (Katona et al., 2012) evaluated two agents in parallel compared to placebo, providing a total of five independent effect sizes to pool, including evaluation of vortioxetine (n = 2) and duloxetine (n = 3). The pooled effect size of both antidepressants (n = 989) versus placebo (n = 616) was 0.24 (95% CI 0.15 to 0.34; *p* < 0.00001), indicative of a small, yet statistically significant, positive effect (Figure 7). Heterogeneity was found to be low, with I^2^ = 0% (*p* = 0.86). Subgroup analysis revealed a pooled SMD, slightly greater for duloxetine (SMD = 0.25) compared to vortioxetine (SMD = 0.24); however, the difference was not statistically significant (*p* = 0.9).

### Pooled Effect Sizes for Direct Comparison Studies (without placebo-controls)

#### SSRIs/SNRIs versus TCAs

**Figure 8.**
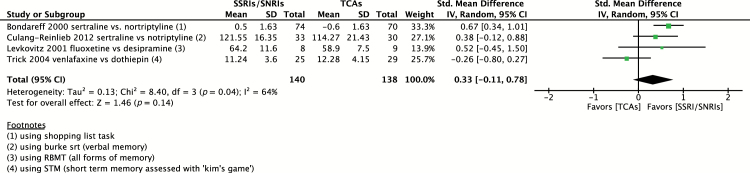
Pooled effect on memory for selective serotonin reuptake inhibitors (SSRI)/selective serotonin and norepinephrine reuptake inhibitors (SNRI) versus tricyclic antidepressants (TCA). CI, confidence interval; RBMT, Rivermead Behavioural Memory Test; SD, standard definition; STM, short term memory.

As summarized in Table 2, four studies were identified comparing SSRIs/SNRIs versus TCAs impact on domains of memory (Bondareff et al., 2000; Levkovitz et al., 2002; Trick et al., 2004; Culang-Reinlieb et al., 2012). No single domain of cognition was consistently tested throughout these four studies, so tests evaluating different domains of memory were pooled. Sertraline (n = 107), fluoxetine (n = 8), and venlafaxine (n = 25) were compared to nortriptyline (n = 100), desipramine (n = 9), and dothiepin (n = 29). The pooled SMD of all SSRIs/SNRIs (n = 140) versus TCAs (n = 138) was 0.33 (95% CI -0.11 to 0.78) in favor of SSRIs/SNRIs; however, the effect was not statistically significant (*p* = 0.14). Heterogeneity was moderate, with I^2^ = 64% (*p* = 0.04). Given that cognitive tests evaluating different domains of memory were utilized, this may have been a cause of heterogeneity. Notably, when removing one study evaluating venlafaxine versus dothiepin (Trick et al., 2004), which appeared to be divergent from the other studies, I^2^ became 0% and the SMD rose to 0.58 (95% CI 0.31 to 0.84; *p* < 0.00001) in favor of SSRIs/SNRIs (Figure 8). Of note, both venlafaxine and dothiepin were dosed twice daily in this study, which negatively affected the quality of sleep (Trick et al., 2004).

**Table 2.** Demographic Information and Study Design of Included Studies Directly Comparing Two Anti-Depressants

Study names	Study Length (weeks)	Diagnostic Criteria	Age (range, Mean ± SD) Sex (%female)	Treatment Group (n)	Cognitive Testing	Favored Treatment
Bondareff et al., 2000	12	-DSM-III -MDD -HAM-D(24) ≥ 18 -Single/recurrent MDE -MMSE ≥ 24	60+ 67.8±6.0 60.0% 67.9±6.6 58.1	Sertraline 50–100mg (74) vs Nortriptyline 25–100mg (70)	WAIS Shopping list task: • Number of items recalled • Number retrieved from long-term recall • Size of list learned • Long-term storage	Favors SSRI *p* = 0.002 Favors SSRI *p* = 0.0001 Favors SSRI *p* = 0.0001 Favors SSRI *p* = 0.02 Favors SSRI *p* = 0.0002
Culang-Reinlieb et al., 2012	12	-DSM-IV -MDD -HRSD(24) ≥ 16 -Single/recurrent MDE -MMSE ≥ 24	45+ 64.85±8.83 61.0% 63.47±8.15 60.0%	Sertraline 50–200mg (33) vs Nortriptyline 1mg/kg (30)	TMT-A TMT-B CPT Purdue Pegboard Buschke SRT Stroop	Neutral NS Neutral NS Neutral NS Neutral NS Neutral NS Neutral NS
Levkovitz et al., 2002	6	-DSM-IV -MDD -HAM-D(17) ≥ 21 -Single/recurrent MDE	25–50 44.5±5.6 50.0% 49.4±5.3 33.33%	Fluoxetine 20mg (8) vs Desipramine 125–200mg(9)	Memory Performance Index: • RBMT • CFT • Paired Associates • Digit Span • DSST	Favors SSRI *p* < 0.02
Trick et al., 2004	26	-DSM-IV -MDD -MADRS ≥ 19 -Single/recurrent MDE	60+ 71.5±7.3 68.89% 71.0±5.7 72.09%	Venlafaxine 75mg (25) vs Dothiepin 75mg (29)	CFF STM CFQ	Favors Venlafaxine *p* = 0.02 Neutral NS Neutral NS
Newhouse et al., 2000	12	-DSM-III -R -MDD -HAM-D(24) ≤ 18 -Single/recurrent MDE -MMSE ≥ 24	60+ 68.0±5.3 63.2% 67.0±5.9 51.3%	Sertraline 50–100mg (80) vs Fluoxetine 20–40mg (80)	DSST	Favors Sertraline *p* = 0.037
Finkel et al., 1999	12	-DSM-III -R -MDD -HAM-D(24) ≤ 18 -MMSE ≥ 24	70+ 74.0±3.6 57.14% 75.0±5.3 48.48%	Sertraline 50–100mg (27) vs Fluoxetine 20–40mg (20)	DSST	Favors Sertraline *p* = 0.0008
Soczynska et al., 2014	8	-DSM-IV -MDD -HRSD(17) ≥ 16 -Single/recurrent MDE	18–50 34.6±9.9 47.4% 41.3±12.9 52.6%	Bupropion XL 150–300mg (19) vs Escitalopram 10–20mg (19)	Memory composite (T score): • Immediate verbal memory • Delayed verbal memory • Immediate nonverbal memory • Delayed nonverbal memory • Working memory	Neutral NS Neutral NS Neutral NS Neutral NS Neutral NS
Gorlyn et al., 2015	8	-DSM-IV -MDD -HRSD(17) ≥ 16 -Single/recurrent MDE	18–65 38.9±11.5 51.9% 36.3±12.5 53.2%	Buproprion 150–450mg (27) vs Paroxetine 25–50mg (30)	Reaction Time Psychomotor Speed Attention Memory Language Fluency Impulse Control	Neutral NS Neutral NS Neutral NS Neutral NS Neutral NS Neutral NS

Buschke SRT, Buschke Selective Reminding Test; CFF, Critical Flicker Fusion; CFQ, Cognitive Failures Questionnaire; CFT, Cognitive Function Test; CPT, Cognitive Performance Test; DSM-III or –IV, Diagnostic and Statistical Manual of Mental Disorders; DSST, Digit Symbol Sign Test; HAM-D, Hamilton Rating Scale For Depression; HRSD, Hamilton Rating Scale For Depression; MADRS, Montgomery-Asberg Depression Rating Scale; MDD, Major Depressive Disorder; MDE, Major Depressive Episode; MMSE, Mini Mental State Examination; RBMT, Rivermead Behavioural Memory Test; SD, standard deviation; SSRI, selective serotonin reuptake inhibitor; STM, Short-Term Memory; TMT-A or -B, Trails Making Test; WAIS, Wechsler Adult Intelligence Scale.

#### SSRIs Versus NDRIs

**Figure 9.**
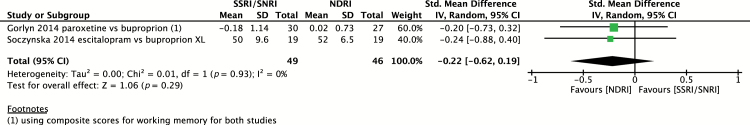
Pooled effect on working memory for selective serotonin reuptake inhibitors (SSRI) versus norepinephrine and dopamine reuptake inhibitors (NDRI). CI, confidence interval; SD, standard definition; SNRI, selective serotonin and norepinephrine reuptake inhibitors.

Two studies were identified directly comparing SSRIs with NDRIs (Soczynska et al., 2014; Gorlyn et al., 2015). Working memory was the only cognitive domain tested in both groups. Escitalopram (n = 19) and paroxetine (n = 30) were compared with bupropion (n = 46). The pooled effect size in favor of bupropion was -0.22 (95% CI -0.62 to 0.19), indicative of no statistically significant difference between groups (Figure 9). Heterogeneity was low, with I^2^ = 0% (*p* = 0.47).

#### Fluoxetine Versus Sertraline

**Figure 10.**
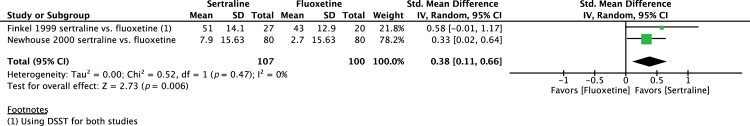
Pooled effect on psychomotor speed (Digit Symbol Sign Test [DSST]) for sertraline versus fluoxetine. CI, confidence interval; SD, standard definition.

Two studies (Finkel et al., 1999; Newhouse et al., 2000) were identified comparing sertraline (n = 107) with fluoxetine (n = 100). Psychomotor speed, as measured by DSST, was the only comparable domain between the two studies. Pooled SMD on psychomotor speed in favor of sertraline was 0.38 (95% CI 0.11 to 0.66; *p* = 0.006), indicative of a medium positive effect (Figure 10). Heterogeneity was low, with I^2^ = 0% (*p* = 0.47).

### Bias of Included Studies

Assessment of bias is summarized in Table 3. All included studies were found to have adequate sequence generation and concealment. Risk of bias for blinded outcome assessment was low in all studies except for one (Levkovitz et al., 2002). Risk of bias based on intention-to-treat analysis was variable between studies as shown in Table 3. Several included studies (Finkel et al., 1999; Bondareff et al., 2000; Newhouse et al., 2000; Ferguson et al., 2003; Trick et al., 2004; Raskin et al., 2007; Culang et al., 2009; Katona et al., 2012; McIntyre et al., 2014; Robinson et al., 2014; Soczynska et al., 2014; Mahableshwarkar et al., 2015) were identified to be high risk of for-profit bias, given that pharmaceutical companies provided funding for these studies.

**Table 3.** Assessment of Bias

Study	Sequence generation	Concealment	Blinded Outcome Assessment	Intention-to- Treat Analysis	For-Profit Bias
Placebo-controlled trials					
McIntyre et al., 2014	Low	Low	Low	Low	High
Katona et al., 2012	Low	Low	Low	Low	High
Mahableshwarkar et al., 2015	Low	Low	Low	High	High
Raskin et al., 2007	Low	Low	Low	Low	High
Robinson et al., 2014	Low	Low	Low	Low	High
Culang et al., 2009	Low	Low	Low	Low	High
Ferguson et al., 2003	Low	Low	Low	Low	HIgh
Georgotas et al., 1989	Low	Low	Low	High	Low
Hoffman et al., 2008	Low	Low	Low	Low	Low
Comparative Trials					
Bondareff et al., 2000	Low	Low	Low	High	High
Culang-Reinlieb et al., 2012	Low	Low	Low	High	Low
Levkovitz et al., 2002	Low	Low	High	Low	Low
Trick et al., 2004	Low	Low	Low	High	High
Newhouse et al., 2000	Low	Low	Low	High	High
Finkel et al., 1999	Low	Low	Low	High	High
Soczynska et al., 2014	Low	Low	Low	Low	High
Gorlyn et al., 2015	Low	Low	Low	Low	Low

Publication bias was assessed using a funnel plot, as shown in Figure 4. A funnel plot was only created for placebo-controlled trials assessing psychomotor speed, as all other forest plots had small numbers of studies and as such a funnel plot would be an inappropriate test. Qualitative assessment of the funnel plot revealed no obvious signs of publication bias; however, the limited number of studies greatly limited the interpretation of the funnel plot. Also of note, an Egger’s test could not be performed, as greater than 10 studies are required for this test to be used according to the *Cochrane Review Handbook.*

## Discussion

The current meta-analysis identified nine placebo-controlled trials assessing the cognitive effects of antidepressants. Pooled effect sizes based on SMD revealed that overall antidepressants have a small positive effect on psychomotor speed and delayed recall; however, the positive effect on cognitive control and executive function was not statistically significant. Other cognitive domains could not be meaningfully assessed due to the lack of comparability of cognitive testing between studies. Of note, the high level of heterogeneity and small number of studies identified in pooling cognitive effects is a major limitation of the current study, which may greatly limit the interpretation of the determined effects. Among the antidepressants assessed under the condition of a placebo-controlled trial, vortioxetine appeared to have the largest effect size on psychomotor speed, executive control, and cognitive control, while duloxetine had the greatest effect on delayed recall.

Subgroup analysis comparing subjects greater than versus less than 65 revealed a greater positive effect in subjects under the age of 65; however, there was no statistically significant difference between age groups. The pathophysiology of cognitive dysfunction associated with MDD may differ in the geriatric population and, as such, a variable effect of antidepressants on cognition may be expected in this group.

Studies directly comparing SSRIs/SNRIs to TCAs were also identified (Bondareff et al., 2000; Levkovitz et al., 2002; Trick et al., 2004; Culang-Reinlieb et al., 2012); however, there was large heterogeneity in cognitive testing, preventing pooling of effect size for a single domain. Domains of memory were thus combined, suggesting SSRIs/SNRIs have a more positive effect on memory compared to TCAs; however, the effect was not statistically significant. A high degree of heterogeneity was identified in this analysis, potentially caused by the pooling of results from different domains of memory. Therefore, the results of this pooled effect size may be invalid; however, cognitive dysfunction secondary to TCA use has long been suggested secondary to the anti-cholinergic effects of TCAs (Baune and Renger, 2014; Bortolato et al., 2014; Keefe et al., 2014).

Two studies (Soczynska et al., 2014; Gorlyn et al., 2015) suggested that SSRIs/SNRIs have an equivalent effect to NDRIs on working memory; however, the pooled effect size was based on a small number of participants and therefore may have been underpowered to detect a difference between these groups.

Sertraline appeared to have a greater effect on psychomotor speed when directly compared to fluoxetine in two separate trials (Finkel et al., 1999; Newhouse et al., 2000). This result alone might not be very clinically relevant; however, it suggests that one should be weary about extrapolating a class effect on cognition based on results from a single drug within that class.

### Limitations

A major limitation of the current meta-analysis was the high level of heterogeneity of cognitive testing used in the identified clinical trials. This heterogeneity in testing greatly limited the comparison and pooling of data. Therefore, the current meta- analysis could not elucidate the relative effect of all antidepressants across disparate cognitive domains and instead was limited to including only a subset of antidepressants for the domains of psychomotor speed, cognitive control, executive control, and delayed recall.

Another limitation of the current study was the moderate level of heterogeneity identified when pooling SMD effect sizes. The heterogeneity may have been caused by the pooling of studies using different antidepressants with different mechanisms of action, including studies with different durations of treatment and different age groups, as shown in Table 1.

Another significant limitation was the highly variable number of subjects pooled for each antidepressant. More specifically, in placebo-controlled trials, vortioxetine and duloxetine were heavily weighted, as these trials had much higher numbers of participants. Therefore, when pooling results for all anti-depressants, the majority of the effect size was determined by the effect of vortioxetine and duloxetine. Further, with removal of vortioxetine from the pooled sample, statistical significance was lost.

Presence of potential bias was identified in most studies, as shown in Table 3. The inclusion of several trials that were industry funded (Finkel et al., 1999; Bondareff et al., 2000; Newhouse et al., 2000; Ferguson et al., 2003; Trick et al., 2004; Raskin et al., 2007; Culang et al., 2009; Katona et al., 2012; McIntyre et al., 2014; Robinson et al., 2014; Soczynska et al., 2014; Mahableshwarkar et al., 2015) presents a significant limitation and potential source of bias to the overall calculated SMDs.

Lastly, a limitation of all studies assessing cognitive function is the gap in understanding the correlation between results of cognitive testing and functional outcomes. While the current study has shown a small positive effect on psychomotor speed and delayed recall as measured by cognitive testing, the precise functional meaning of this remains largely unknown (Baune et al., 2010; Bortolato et al., 2014).

## Conclusion

Due to the known persistence of cognitive dysfunction during remission (Bora et al., 2013) and demonstrated small positive effect of antidepressants on delayed recall and psychomotor speed, the investigation of other cognitively enhancing agents to be used adjunctively to current antidepressants in the MDD population is merited.

The current study also elucidated the large difficulties appropriately comparing cognitive clinical trials due to the currently high level of heterogeneity of cognitive testing. Therefore, improved standardization of cognitive testing with efforts made to evaluate every domain separately in every study would be greatly beneficial. As well, a combination of both self-report and objective cognitive testing may aid in the understanding of subjective cognitive complaints in MDD.

Future studies using cognitive function as a pre-specified primary outcome are needed, as the majority of studies discussed were evaluating cognition as a secondary outcome. As well, studies should include placebo controls due to the expected improvement in cognitive testing seen with repeat testing (e.g. practice effect). Adequate statistical testing to allow for path analysis, and thus determination of direct and indirect effects of antidepressants on cognition, should be considered for future studies.

## Statement of Interest

Drs Rosenblat and Kakar have no conflicts of interest. Dr McIntyre has received research grant support from Lundbeck, Astra Zeneca, Pfizer, Shire, Otsuka, Bristol Myers Squibb, National Institute of Mental Health, Stanley Medical Research Institute, Canadian Institutes for Health Research, and the Brain and Behavior Research Foundation. Dr McIntyre has also received speaker/consultant fees from Lundbeck, Pfizer, Astra Zeneca, Elli Lilly, Janssen Ortho, Sunovion, Takeda, Forest, Otsuka, Bristol Myers Squibb, and Shire.

## Supplementary Material

Supplementary Material
