# Supplementary material for: The Cognitive Effects of Antidepressants in Major Depressive Disorder: A Systematic Review and Meta-Analysis of Randomized Clinical Trials
Source: Int J Neuropsychopharmacol. 2015 Jul 25;19(2):pyv082. doi: 10.1093/ijnp/pyv082 (PMC4772818; doi:10.1093/ijnp/pyv082)
Supplement: Supplementary Material [file Supplementary_Material.docx]

**Supplementary Material**

**Additional search terms used for identifying additional articles:** major depressive disorder, unipolar depress*, neuropsychology*, neurocognit*, cognitive, cognition, impairment, deficit, function, antidepressant, SSRI, SNRI, tricyclic, TCA, sertraline, citalopram, escitalopram, vortioxetine, paroxetine, fluoxetine, fluvoxamine, duloxetine, venlafaxine, desvenlafaxine, levomilnacipran, vilazadone, reboxetine, mirtazapine, trazadone, amitriptyline, clomipramine, desipramine, doxepin, nortriptyline, trimipramine.

**Supplemental table 1: Summary of notable excluded studies**

| **Study** | **Agent** | **Description** | **Reason for exlusion** |
| --- | --- | --- | --- |
| **Uher et al. (2009)** | Escitalopram, nortriptyline | A partly randomized open label study showing greater improvement in cognitive measures (overall cognitive effect reported, no domain specific reporting) from escitalopram compared to nortryptyline, in fitting with the results of other studies reviewed (Bondareff et al., 2000; Levkovitz et al., 2002; Trick et al., 2004; Culang-Reinlieb et al., 2012) | Open label study |
| **Nebes et al. (2003)** | Nortriptyline, paroxetine | For both nortriptyline and paroxetine, cognitive function improved over the 12-weeks of treatment, however, cognitive scores were still significantly lower than healthy controls, even in patients who achieved full remission. | Healthy controls were used instead of MDD placebo-controls or direct comparison of the two agents |
| **(Herrera-Guzman et al., 2009; Herrera-Guzman et al., 2010a; Herrera-Guzman et al., 2010b)** | SSRIs, SNRIs | In two studies analyzing one data set, Herrera-Guzman et al. evaluated the effects of SSRIs and SNRIs on various domains of cognition in MDD patients. Cognitive function never reached the level of healthy controls; however, both SSRI and SNRI treatment improved all domains of cognition measured (attention, executive function, memory and processing speed) independent of treatment response (e.g. remission of depressive symptoms). Of note, other studies reviewed also suggested that improvement in cognition was independent of treatment response (Georgotas et al., 1989; Culang-Reinlieb et al., 2012). Conversely, Culang et al. (2009) found that the cognitive benefits of citalopram were dependent on antidepressant response. | Excluded from quantitative analysis, as they were not reported as randomized trials. |
| **Allain et al. (1992)** | moclobemide, viloxazine and maprotiline | Allain et al. evaluated the cognitive effects of moclobemide, viloxazine and maprotiline showing improvement in attention and memory from moclobemide use and not with viloxazine or maprotiline | Agent being investigated was either not generally available and or because the mechanisms of action did not principally involve the monoaminergic system. |
| **Austin et al. (2000)** | Apomorphine | Apomorphine, a dopamine agonist, was shown to have no beneficial effect to any domain of cognition | Same as above |
| **Nickel et al. (2003)** | Tianeptine, paroxetine | Cognitive assessments showed no significant differences between  the two drugs investigated | Primary mechanism of action of tianeptine is glutamate modulation rather than via the monoamine system (historically categorized as a TCA) and was therefore excluded |
| **Jeon et al. (2014)** | Tianeptine, escitalopram | Both groups significantly improved in subjective cognitive impairment in memory and concentration.  Mixed effects model repeated measures analyses revealed that the tianeptine group had a significant improvement in scores of commission errors and verbal immediate memory  from baseline to 12 weeks, compared with the escitalopram group, after controlling for age, sex, education years, baseline scores, and changes of depression severity. | Same as above |
